# Strategies for structuring interdisciplinary education in Systems Biology: an European perspective

**DOI:** 10.1038/npjsba.2016.11

**Published:** 2016-05-26

**Authors:** Marija Cvijovic, Thomas Höfer, Jure Aćimović, Lilia Alberghina, Eivind Almaas, Daniela Besozzi, Anders Blomberg, Till Bretschneider, Marta Cascante, Olivier Collin, Pedro de Atauri, Cornelia Depner, Robert Dickinson, Maciej Dobrzynski, Christian Fleck, Jordi Garcia-Ojalvo, Didier Gonze, Jens Hahn, Heide Marie Hess, Susanne Hollmann, Marcus Krantz, Ursula Kummer, Torbjörn Lundh, Gifta Martial, Vítor Martins dos Santos, Angela Mauer-Oberthür, Babette Regierer, Barbara Skene, Egils Stalidzans, Jörg Stelling, Bas Teusink, Christopher T Workman, Stefan Hohmann

**Affiliations:** 1 Department of Mathematical Sciences, Chalmers University of Technology and University of Gothenburg, Gothenburg, Sweden; 2 Division of Theoretical Systems Biology, German Cancer Research Center (DKFZ), Heidelberg, Germany; 3 Centre for Functional Genomics and Bio-Chips, Institute of Biochemistry, Faculty of Medicine, University of Ljubljana, Ljubljana, Slovenia; 4 University of Milano-Bicocca, Department of Biotechnology and Biosciences, Milano, Italy; 5 Department of Biotechnology, NTNU – Norwegian University of Science and Technology, Trondheim, Norway; 6 Department of Informatics, Systems and Communication, University of Milano-Bicocca and SYSBIO Centre of Systems Biology, Milano, Italy; 7 Department of Marine Sciences, University of Gothenburg, Gothenburg, Sweden; 8 Warwick Systems Biology Centre, University of Warwick, Warwick, UK; 9 Department of Biochemistry and Molecular Biology, Faculty of Biology, University of Barcelona, Barcelona, Spain; 10 CNRS, Paris, France; 11 Imperial College London, London, UK; 12 University College Dublin, Dublin, Ireland; 13 Laboratory for Systems and Synthetic Biology, Wageningen UR, Wageningen, Netherlands; 14 Universitat Pompeu Fabra, Department of Experimental and Health Sciences, Barcelona, Spain; 15 Unité de Chronobiologie Théorique, Faculté des Sciences, CP 231 and Interuniversity Institute of Bioinformatics in Brussels (IB)2, Université Libre de Bruxelles, Brussels, Belgium; 16 Theoretical Biophysics, Humboldt-Universität zu Berlin, Berlin, Germany; 17 SystemsX.ch, Zurich, Switzerland; 18 LifeGlimmer GmbH, Berlin, Germany; 19 University of Heidelberg, Heidelberg, Germany; 20 BioQuant Center, University of Heidelberg, Heidelberg, Germany; 21 Wageningen UR, Netherlands; 22 Institute of Microbiology and Biotechnology, University of Latvia, Riga, Latvia; 23 ETH Zurich, Zurich, Switzerland; 24 Vrije Universiteit Amsterdam, Amsterdam, Netherlands; 25 Technical University of Denmark, Copenhagen, Denmark; 26 Department of Biology and Bioengineering, Chalmers University of Technology, Göteborg, Sweden

## Abstract

Systems Biology is an approach to biology and medicine that has the potential to lead to a better understanding of how biological properties emerge from the interaction of genes, proteins, molecules, cells and organisms. The approach aims at elucidating how these interactions govern biological function by employing experimental data, mathematical models and computational simulations. As Systems Biology is inherently multidisciplinary, education within this field meets numerous hurdles including departmental barriers, availability of all required expertise locally, appropriate teaching material and example curricula. As university education at the Bachelor’s level is traditionally built upon disciplinary degrees, we believe that the most effective way to implement education in Systems Biology would be at the Master’s level, as it offers a more flexible framework. Our team of experts and active performers of Systems Biology education suggest here (i) a definition of the skills that students should acquire within a Master’s programme in Systems Biology, (ii) a possible basic educational curriculum with flexibility to adjust to different application areas and local research strengths, (iii) a description of possible career paths for students who undergo such an education, (iv) conditions that should improve the recruitment of students to such programmes and (v) mechanisms for collaboration and excellence spreading among education professionals. With the growing interest of industry in applying Systems Biology approaches in their fields, a concerted action between academia and industry is needed to build this expertise. Here we present a reflection of the European situation and expertise, where most of the challenges we discuss are universal, anticipating that our suggestions will be useful internationally. We believe that one of the overriding goals of any Systems Biology education should be a student’s ability to phrase and communicate research questions in such a manner that they can be solved by the integration of experiments and modelling, as well as to communicate and collaborate productively across different experimental and theoretical disciplines in research and development.

## Introduction

Systems Biology aims at the elucidation of the biological properties that emerge from the interaction of genes, molecules, cells, tissues and organisms.^1–3^ Hence, the Systems Biology approach addresses the now well-established fact that many relevant properties in biology and medicine are not ruled by single genes. For instance, predisposition for many human diseases or desired properties of crops, farm animals and microorganisms are determined by the interplay of numerous genes and their products in complex networks. The properties of those networks can commonly not be predicted on the basis of properties of the individual components, such as proteins. Rather, elucidation of the network properties and how they affect biological function requires specific types of often multilayered data sets as well as mathematical models coupled with computational tools to rationalise the network properties and predict the effect of system perturbations.^4^

In addition, Systems Biology investigates other emergent system's properties such as, e.g., the functional importance of dynamics, the information processing in networks and the impact of spatial processes on cellular function. All of these aspects again have in common that they cannot be derived from the study of single genes or proteins, but need an understanding of their quantitative relationship, their interaction and their distribution in space, all of which necessitate the usage of mathematical models as well as precise experimentation.

The European Systems Biology community presently comprises more than 7,000 researchers (there are roughly 500,000 researchers within life sciences in Europe^5^) working in more than 800 European research institutions (European Systems Biology Community website, community.isbe.eu). Only ~15% of these institutions are specifically dedicated to Systems Biology, whereas the rest are either generalists (e.g., universities) or organisations placed within different areas of the life sciences (such as microbiology, plant physiology and medicine). European in Systems Biology initially focused in basic pilot studies on the development and use of predictive models, primarily at the molecular level, such as metabolic networks. More recently, there is a growing effort towards the implementation of systems approaches within biomedical research (or ‘systems medicine’) and across the biotech industry.^6^ Recent examples include the transnational programmes Coordinating Action Systems Medicine, the ERANet projects ERACoSysMed and ERASys*APP* and EC programmes within the Bio-Based Industries or the Innovative Medicines Initiative funding scheme. Current research directions also include the use of multiscale modelling, which aims at understanding the functioning of complete organs and organisms as the result of the dynamic interplay in time and space of molecules, cells and tissues. Such integrative efforts are supported by translational EC programmes to generate robust data infrastructure and exchange standards for the different omics technologies (e.g., Coordination of Standards in Metabolomics (COSMOS, http://www.cosmos-fp7.eu/) or the HUPO Proteomics Standards Initiative (PSI, http://www.psidev.info/) and data integration and modelling itself developed by the COMBINE community (http://co.mbine.org)). These initiatives are already creating a strong basis for new avenues in biomedical and pharmaceutical research. Examples include the Virtual Human Heart, the Virtual Liver Network (http://www.virtual-liver.de/), the Human Metabolic Atlas (http://www.metabolicatlas.org/) and projects in the frame of the international Virtual Physiological Human programme (http://www.vph-institute.org/) and Physiome Project (http://physiomeproject.org).

The systems approach to biology is now widely accepted and becomes part of mainstream biology, medicine and bioengineering. Consequently, Systems Biology is now also part of the educational portfolio at many universities either as dedicated programmes or as part of the genomics and bioinformatics curricula.^7–12^ However, the scope and content of those programmes differ very widely, making it difficult for employers and students alike to determine into which type of employment, role or career path Systems Biologists would fit. Our aim is to suggest an improved structure for the Systems Biology education landscape and provide common grounds to facilitate education and career planning for students and for employers to recruit Systems Biologists into appropriate positions. With ‘education’ we refer to courses and programmes offered by universities and similar higher education institutes. In Europe, those are nowadays generally structured into 3-year Bachelor programmes and 2-year Master’s programmes. We reserve the term ‘training’ for activities that are offered outside such ‘education’ programmes in specialised courses or as life-long professional training. University education is arranged in different ways in different countries, or even at different universities within a country. For instance, courses may be offered in parallel over an entire semester or academic year (school type) or as blocks of a certain number of weeks, or a mix thereof. Educational programmes may economically and administratively be managed by departments, faculties or universities, where the former seems to be the most common situation.

Interdisciplinary education in general and within the life sciences and Systems Biology in particular is facing different obstacles that are summarised below:

- Education is organised according to disciplines/departments at many higher education institutes. The fact that departments ‘own’ the educational programmes and the financial resources to arrange them directly counteracts interdisciplinary education. A more appropriate model would allocate resources directly to programmes that distribute tasks and resources in accordance with the needs for interdisciplinary education.
- At many institutions all of the appropriate and relevant competences in experimental and theoretical sciences may not be available. Exchange of staff or availability of online courses may be approaches to solve such issues.^12^
- Students may be poorly prepared or not be aware of the systems approach to biology because high school and Bachelor programmes may not touch upon those. Existing biology education is traditionally not quantitative, whereas this is a hallmark of the systems-level approach. The Systems Biology community certainly needs to work on penetrating high school and Bachelor levels of education as well to raise awareness early on.
- There is a lack of example or model curricula that could guide institutions that either want to set up a new programme or wish to benchmark their existing programme. This topic is the main focus of the present article.
- Although some general literature references can be effectively exploited for education in Systems Biology,^13–15^ there is a general lack of comprehensive textbooks and teaching material able to adequately cover all topics in a complete interdisciplinary programme in Systems Biology.

We collected experts in Systems Biology and education performers at two workshops held in Heidelberg and Gothenburg during fall 2014 and spring 2015, respectively. Our discussions resulted in proposals for (i) a catalogue of generic skills and competences that Systems Biology students should have acquired in relevant programmes, (ii) educational topics and approaches that could lead to acquisition of those skills and (iii) possible career paths for Systems Biologists. Here we present a reflection of the European situation and expertise, which we hope will be useful internationally. We trust that those concepts will help lifting the status and impact of existing programmes, establishing new programmes where Systems Biology is not yet part of the portfolio, recruiting students and teachers to such programmes, shaping Systems Biology careers and promoting collaborations between programmes.

### General skills that students should have acquired in a Systems Biology educational programme

It is essential to define the competences that students should have acquired in a Systems Biology educational programme; on the one hand for the students to better anticipate potential career paths after having completed education, and for potential employers in academia and industry to match candidate skills with their needs. It may be difficult to define those skills and competences down to details of, for instance, types of modelling approaches and experimental techniques, simply because there are too many. Therefore, we suggest ‘higher level’ skills, that rather reflect the interdisciplinary systems approach and its way of thinking and operating. We suggest that graduates from Systems Biology Master’s programme should have acquired the following skills:

- An understanding of the type of biological, medical or bioengineering questions that can be approached by integrating experimental data sets with mathematical modelling.
- Capacity of formulating research problems such that they can be solved by an integrated experimental/mathematical approach.
- A good appreciation of the Systems Biology iterative cycle: modelling, prediction and experimental verification.
- A well-developed ability to communicate scientific questions across experimental and theoretical disciplines and to collaborate across disciplinary borders.
- A good awareness of different types of modelling and their applicability to research problems as well as in-depth understanding and hands-on experience in specific mathematical modelling approaches.
- A good awareness of the type of data generation and modelling approaches that are suitable for a given research problem as well as in-depth understanding and hands-on experience in specific experimental techniques.
- Skills in data handling, management and visualisation, including an understanding of statistical analyses suitable for different types of data and experimental designs.
- An ability to critically assess evidence and scientific argumentation in integrative studies of biological systems based on an understanding of both experimental and theoretical/computational biology methodologies.

### Basic educational curriculum with flexibility to adjust to different application areas and local research strengths

In order to build basic educational programmes, we decided not to propose a list of courses, simply because different institutions most likely have different curriculum structures. Therefore, suggesting specific courses may be too rigid and hence limit the possibility to apply our recommendations, for instance, within already existing programmes. Instead, we have divided Systems Biology education into different areas and have broken down those into topics that we believe should be covered by any programme in the field (Table 1). The list does not outline the depth to which the topic should be taught because this may be very different for different types of students. For instance, a student with an experimental biology background should master many or all of the topics under ‘Experimental design, measurement, analysis, interpretation and knowledge generation’, while she/he probably should have a principle understanding of ‘Mathematical and computational concepts’ but might not develop into the specialist to perform modelling. In other words, in order to fulfil the criteria listed above, students should be true experts/specialists in some of the areas below while having sufficient knowledge of the other areas to efficiently communicate and collaborate with an expert/specialist in another relevant field. The specific courses will be tailored as a function of the students profile and the expertise available at the University.

### Means and methods for education

We believe that a successful interdisciplinary programme must be more than a collection of discipline-specific courses combined in a curriculum. Instead, ideally all courses should combine input from different disciplines. For instance, biological phenomena should be taught from both an experimental and theoretical perspective at the same time. Although this appears obvious, it can be ambitious in practice as it puts high demands on teachers and students alike. However, it is our experience that the most rewarding courses are those where students from different backgrounds jointly develop concepts and solve challenging tasks.^7–12^ Therefore, we recommend that (i) programmes ideally should include students from different backgrounds (with certain entry skills, see below), (ii) courses should keep those students together wherever possible and (iii) much of the education should be problem-oriented rather than teaching facts and concepts disconnected from research questions.^16–18^ This of course is only possible at a level where students have strong foundations in at least one discipline, i.e., at Master’s level. This said, ambitious bachelor-level programmes, such as that given at the Princeton’s Lewis Sigler Institute (http://lsi.princeton.edu/integratedscience/), Universitat Pompeu Fabra in Spain (https://portal.upf.edu/web/etic/bachelor-degree-biomedical-engineering) or at the National Autonomous University of Mexico (http://www.lcg.unam.mx/about) start from day 1 with integration of experiment and theory. However, it is important to emphasise that these are undergraduate-level programmes with rather different set-up than general master’s degree curriculum.

The following aspects were discussed by our group:

- Course content should generally and, where possible, adopt an integrated approach to learning, starting with, for example, small (dynamic) systems to introduce basic experimental and theoretical concepts.
- ‘Sherlock Holmes practicals’, i.e., challenging exercises, where students from different backgrounds jointly work around a given project/problem, are important elements in any programme-fostering creativity, communication and collaborative skills.^8^
- Participation to the International Genetically Engineered Machine (iGEM) competition (http://igem.org/)^19–21^ should be encouraged.
- Assessment of student’s performance should ideally combine both individual and group performance, where groups consist of students with different backgrounds.
- In many instances, although probably not always, it is possible to teach modelling approaches by applying them to a biological problem/system.
- Programming/modelling tools as well as modelling standards should be taught at an early stage in the programme and used throughout.
- If possible, pure lecture or pure practical courses should be avoided and both types should be integrated, demonstrating the core of Systems Biology and that it is about a close collaboration of experimentalists and theoreticians working on the same topic.
- Case studies (primary literature articles) are excellent tools to demonstrate how skills can be applied to different biological systems and how new knowledge is being generated; classical papers may illustrate the history/development of the field.
- Internships/projects/Master thesis should ideally integrate experimental and theoretical approaches but not generally force such project designs on students.

Course content should in a maximal manner build on each other regardless of different set-ups for programmes run by different organisations, i.e., semester-long parallel courses or blocks of different length running consecutively. In cases where the course structure deviates significantly from the ‘standard’ European Bologna concept, we advise that the course content should be given in a modular way such that the course length in different systems will not be a hurdle.

### Entry skills for Systems Biology programme

A major challenge for any interdisciplinary educational programme concerns the background or entry skills of the students. It is imperative that master’s students have different backgrounds and a strong foundation in at least one relevant discipline, in particular that they have acquired a language and a way of thinking of that scientific field. For Systems Biology programmes, students with Bachelor background in Biology, Biotechnology, Medicine, Physics, Chemistry, Mathematics, Engineering and Computer Science could be considered. This means that the programmes will be entered by students who should have skills in experimental or theoretical sciences, but at the same time may have very limited knowledge of biology or mathematical modelling. It, therefore, may be necessary to introduce students before or at the beginning of the programme to basic principles in biology or mathematics. Table 2 provides a prerequisite for a Systems Biology graduate programme summarizing main concepts that are necessary to ‘level-up’ students in order to successfully complete the programme. In our experience, the majority of students entering master’s programmes in Systems Biology are coming from either experimental or theoretical background. In cases where students will not strictly fit to either of the categories, the flexibility of the programme will allow these students to learn specific skills they are missing. Later in the course, these notions will be revisited, deepened and applied in specific contexts.

### Conditions that should improve the recruitment of students to such programmes

Recruitment of students to interdisciplinary programmes can be a challenge for a number of reasons, some of which we try to address with this position paper. It will be important to further outline, in collaborations between academic and private-sector recruiters, the career paths for students who have undergone interdisciplinary training, as compared with those who focused on a single discipline. We have experienced that indeed such students are in demand, especially for PhD programmes internationally, but of course also industry must be aware of the skills of Systems Biology students. This can be facilitated by arranging internships or inviting industry representatives to participate in the lectures and seminars. The Biotechnology and Biological Sciences Research Council (BBSRC) and the Medical Research Council (MRC) have recently released an important paper stressing the need for interdisciplinary skills.^22^ The Association of the British Pharmaceutical Industry (ABPI) in the 2015 report highlights the demands for bioinformaticians and systems biologists. ABPI report is based on research from 93 industry leaders from 59 organisations, revealing that the most concerning skill gaps are in the interdisciplinary areas involving mathematics and biology, which are essential for the development of the personalised medicines.^23^

Another problem for recruiting students to interdisciplinary programmes is the lack of exposure to cross-disciplinary approaches in high school and Bachelor programmes. Teachers and instructors need to be trained in interdisciplinary biology. System-level approaches and the systems' way of thinking need to penetrate high school and Bachelor programmes. The multidisciplinary character of high school programmes, in particular, makes it especially possible to emphasise such type of systems-level thinking among their young students. We must raise interest among pupils and Bachelor students in the opportunities and challenges of operating across disciplines to solve the biological, medical and bioengineering challenges of the Twenty first century. Therefore, Systems Biology must urgently reach out to the education of young people at schools and at Bachelor level. This can be achieved in the following ways:

- Systems Biology must become part of teacher education programmes such that they are trained to teach pupils. This also means that modern high school textbooks in Biology and Mathematics should contain sections on those topics and emphasise the importance in biological research of integration between experimental and theoretical sciences.
- Systems Biology must become part of the life-long training of high school as well as University teachers.
- Many universities entertain outreach programmes to high schools as part of their student recruitment strategy. Genomics, Bioinformatics and Systems Biology must be part of those outreach initiatives, which can be in the form of lectures at schools or pupil-visit-the-university activities.
- Systems Biology elements need to become part of the high school programmes in Sciences, in particular within Biology, Mathematics and Computer Sciences.
- Systems Biology elements need to become part of Bachelor programmes even if those may be discipline-oriented. At the minimum, those activities should be sufficient as teasers to attract students to subsequent interdisciplinary Master’s programmes.

### Career paths for students who underwent a Systems Biology education

Today, there is rapidly growing demand for Master students with a Systems Biology profile both in industry and academia, offering them a broad spectrum of job opportunities, particularly in the biomedical field.^24–27^ Such students should also have a competitive advantage in environments where flexibility, critical thinking and the ability to operate across disciplinary borders are important.^28^ At the same time, they may compete less well for jobs where interdisciplinarity is less relevant. This is because of the fact that they may not have acquired the same depth within a given field because they have taken courses covering other disciplines. We believe that this disadvantage will rapidly be compensated for by an increasing number of opportunities where flexibility and interdisciplinarity are desired. Therefore, it is important for employers to understand the skills and competences that Systems Biologists have acquired in their education. A systems biologist will be able to employ and analyse large/complex data sets and to interpret the results with a critical mind set. It is important to emphasise that independent of the life science field—methods, tools, approaches that are learnt by the students within Systems Biology programme are a horizontal expertise and can easily be transferred to other life science fields as well. In the following, we list areas in which we believe that students completing a Systems Biology programme will find future jobs and opportunities to further develop in their professional careers.

- *Academia* is increasingly moving into interdisciplinary recruitments. Many medical or biological schools have been recruiting bioinformaticians and statisticians over the last 20 years and establishing Systems Biology research and educational programmes.^22,23^
- *Research-based business spin-offs*, for instance, within Synthetic Biology and Industrial Biotechnology, require highly skilled work force in research but also management. Numbers of research-intense smaller companies are increasing, although the overall job market is relatively small and demands a high degree of flexibility, in particular with respect to the location of the job.^29–31^
- *Biotechnology and bioengineering companies* of different sizes are also recruiting skilled staff with an interdisciplinary background. This includes companies active in the biofuel sector, fine and bulk chemical companies, the agro- and food biotechnology sector as well as actors within bioremediation.
- Larger *research-active pharmaceutical companies* are increasingly applying Systems Biology approaches in drug discovery, development of diagnostics and *in silico* clinical trials. Research may also be performed in collaboration with universities, research institutes or spin-off companies that hold relevant intellectual property. Although the move towards system-level approaches, and hence the demand for skilled work force, does not seem to progress as fast and as massively as was anticipated 10 years ago, companies do recruit professionals with interdisciplinary background.^32–35^
- *Data analysis sectors* (banks, insurance, consultancy and policy making) should certainly be interested in persons with an interdisciplinary background and a wide scope of expertise, rather than narrow specialists.
- *The public health sector*, especially within monitoring and analysis, should also benefit from work force with both medical and computational skills.
- *The public sector of environmental monitoring and sustainable and predictive resource management* is another area where persons with an interdisciplinary background and skills in data management and handling should find employment.
- *Science and research management*, both private and public, certainly is an area where broader scope and experience from different disciplines is more in demand than a high degree of specialisation.
- *Scientific communication*, scientific writing for lay persons. The cross-discipline communication skills acquired by working in a Systems Biology context will give those trained in systems biology an advantage over the disciplinary specialists.

Generally, all those employment opportunities are not fundamentally different from those for the specialists, but we believe that in many instances the interdisciplinary background may offer an advantage for the employer, such as facilitates adaptability to different scenarios in changing business areas and the skills to make appropriate future predictions. Usually, employment opportunities increase significantly when persons are not restricted to a geographic region or country, given that the overall market is not very large at any given location.

### Mechanisms for collaboration and excellence spreading among education performers in Europe and globally

There is a clear need for closer collaboration and exchange between Systems Biology education performers. This will be relevant in order to harmonise programmes along the lines described here, but also to develop curricula further and to make expertise available via exchange of students and teachers.

There are different ways how such exchange can be achieved. The ERASys*APP* project, under the leadership of Systems-X.ch in Switzerland, set up web portals that list educational programmes (https://www.erasysapp.eu/training-and-exchange/graduate-study-programs), and also provide education material (http://www.sbedu.eu/). These tools are extremely useful when planning new programmes or setting up courses.

There are funding mechanisms available within the EC framework programme that could also help, and are partially aimed at improving the collaboration between educational programmes, for instance, within the Marie Skłodowska-Curie actions (http://ec.europa.eu/research/mariecurieactions/) or the Eramus+ programme (http://eacea.ec.europa.eu/erasmus-plus_en). We trust that the work presented here will contribute to harmonising Systems Biology education and stimulate exchange of material, teachers and students.

## Figures and Tables

**Table 1:** Suggested basic core areas for Systems Biology education

**Masters level programme in Systems Biology**
*Suggested basic core areas for Systems Biology Education*
*Mathematical and computational framework*
Linear algebra—as relevant for stoichiometric modelling, genome-scale metabolic reconstructions and models; stability analysis—as relevant for stability of genetic circuits
Nonlinear dynamics—as relevant for signalling cascades, kinetic metabolic models, pattern formation, enzyme dynamics, cell differentiation and cellular decision-making
Stochastic modelling—as relevant for gene expression circuits, cell motility, ion channels, protein–protein interaction, diffusion and signal transduction pathways
Spatial modelling—as relevant for morphogenesis, cell communication, tissue formation, crowding, biofilms
Control theory—as relevant for design and analysis of metabolic pathways, gene expression circuits, pharmacokinetics and pharmacodynamics
Discrete and logic models—as relevant for genetic networks, signalling networks and cellular differentiation
Complex network analysis—as relevant for metabolic networks, protein–protein interaction networks, gene–disease networks
Optimisation—as relevant for metabolic engineering, genome-scale metabolic models, parameter estimation in signal transduction networks and reverse engineering

*Networks and processes of life*
Metabolic networks—as relevant for physiology, human diseases and metabolic engineering; fluxes, kinetics, rates and stoichiometry
Signalling networks—as relevant for information processing and engineering of cells and organisms; dynamics, feedbacks and adaptation
Gene regulation networks—as relevant for cellular decision making and differentiation, bi- and multistability phenomena as well as circuit design in Synthetic Biology
Cell and population networks—as relevant for development, pattern formation, disease (especially cancer), infection and ecology; cell variability phenomena
Genetic networks—as relevant for multifactorial traits and diseases, epistasis, as well as genome-wide association studies and meta-analysis thereof
Protein (and other types of) interaction networks—as relevant for complex inference and functional modules
Oscillatory processes—as relevant for cell cycle, circadian rhythms, metabolic oscillations and other processes where timing regulates states of functional activity/inactivity

*Scientific programming*
Programming: e.g., Matlab or Mathematica, Python, Perl, Java, R, C and C++
Tools for genome-scale metabolic models, kinetic modelling (stochastic and deterministic), network analysis: e.g., Copasi, Cytoscape, OptFlux, XPP-Auto and COBRA
Standards: e.g., SBML, SBGN and MIRIAM
Methods and software tools for biological data visualisation

*Bioinformatics and statistics*
Fundamentals of DNA, RNA and protein sequence analysis
Integrative bioinformatics—interoperability, ontologies, semantics, databases and standards
Genomics of communities, meta-genomics of populations of cells and organisms
Molecular evolution, phylogeny and population genetics
Complex genotype–phenotype relationships—genome-wide association studies for human diseases and desirable traits in plants, animals and microbes
Data analysis—standard algorithms, basics of supervised and unsupervised statistical learning, data integration
Statistical inference—use of appropriate statistical tests, reverse engineering
Machine learning—clustering; neural networks, random forest

*Experimental design, measurement, analysis, interpretation and knowledge generation*
Quantitative imaging/microscopy—single-cell analysis using flow cytometry or microscopy or spectroscopy, image analysis and quantification, cell variability analysis
Global and high-throughput data—genetic, transcriptome, proteome and metabolome
Biochemical *in vitro* and *in vivo* assays for quantitative properties of proteins, reactions and interactions
Handling and culturing of cells and organisms
Quantitative and time-resolved experimental methods at low throughput—levels and modifications of biomolecules, especially proteins and RNAs
Principles of system perturbations—genetic, experimental, pharmacological perturbations to test, challenge and control systems
Principles of systems engineering—*in vitro* systems design, *in vivo* implementation employing genetic and biological engineering as well as evolutionary approaches, testing and optimisation of designed circuits

(i) Mathematical and computational framework; (ii) networks and processes of life; (iii) scientific programming, (iv) bioinformatics and statistics; and (iv) experimental design, measurement, analysis, interpretation and knowledge generation. Each area is broken down into specific topics and the relevance in which it should be taught.

**Table 2:** Skills that should be introduced to students entering a Systems Biology Programme from either an experimental (left) or theoretical (right) background

*Introductory module*	
*Experimental background*	*Theoretical background*
*Entry skills*	
Linear algebra: matrix representations of linear equations, matrix analysis, vector spaces, linear dependence/independence, linear combinations and spans, basis, eigenvalue and eigenvectors, linear transformations	The cell concept and cell organisation: cell diversity, prokaryotes and eukaryotes, organelles, cytoskeleton and cell structure
	
Differential and integral calculus: limits, derivatives, derivative properties, power rule, mean value theorem, integrals, fundamental theorem of calculus, area under the curve, series, sequences,	Basic and quantitative biochemistry and physiology: macromolecules, the function of membranes and membrane structure, what drives life: basics of thermodynamics, bioenergetics, metabolic pathways, catabolism and anabolism, basic protein structures, protein properties: enzymes, antibodies, affinities
	
Differential equations: first-order differential equations, directions fields, existence and uniqueness of solutions, separable equations, Laplace transformation	Genetics and inheritance: genomes and chromosomes, epigenetics, genetic variation, DNA replication and repair, viruses and transposable elements
	
Basics of graph theory and Boolean logics	Gene expression: from DNA to RNA: transcription, from RNA to proteins: translation, gene regulation
	
Structured programming, pseudo-code, data structures, flow diagrams	Cellular responses: receptors and signalling, cell division, apoptosis and cell death, protein sorting, protein secretion
	
Numerical analysis and algorithms: interpolation and numerical approximation of functions, methods for integration of functions and for solving ordinary and partial differential equations, methods for solving linear algebra problems, methods for solving systems of linear equations, Monte Carlo techniques, stochastic simulation algorithms	Multicellularity: the function of tissues, hormones, development, causes of disease
	
Multivariate calculus: double and triple integrals, volume under a surface, partial derivatives, gradients, divergence, surface integrals	Evolution: selection and adaptation, basic population genetics
	
Introduction to programming and algorithmics	Techniques: genetic model organisms, DNA technology and sequencing, DNA microarray
